# Investigation of the Charge-Transfer Between Ga-Doped ZnO Nanoparticles and Molecules Using Surface-Enhanced Raman Scattering: Doping Induced Band-Gap Shrinkage

**DOI:** 10.3389/fchem.2019.00144

**Published:** 2019-03-19

**Authors:** Peng Li, Xiaolei Wang, Xiaolei Zhang, Lixia Zhang, Xuwei Yang, Bing Zhao

**Affiliations:** ^1^State Key Laboratory of Supramolecular Structure and Materials, College of Chemistry, Jilin University, Changchun, China; ^2^College of Chemistry, Jilin University, Changchun, China

**Keywords:** charge-transfer, Ga-doped ZnO, SERS, 4-MBA, band gap shrinkage

## Abstract

Semiconductor nanomaterial is a kind of important enhancement substrate in surface-enhanced Raman scattering (SERS), and the charge-transfer (CT) process contributes dominantly when they are used as the enhancement substrate for SERS. Doping has significant effect on the CT process of semiconductor nanomaterials. Yet till now, none attempts have been made to explore how doping affects the CT process between the semiconductor and probe molecules. For the first time, this paper investigates the effect of gallium (Ga) doping on the CT process between ZnO nanoparticles and 4-mercaptobenzoic acid (4-MBA) monolayer. In this paper, a series of Ga-doped ZnO nanoparticles (NPs) with various ratio of Ga and Zn are synthesized and their SERS performances are studied. The study shows that the doped Ga can cause the band gap shrinkage of ZnO NPs and then affect the CT resonance process form the valence band (VB) of ZnO NPs to the LUMO of 4-MBA molecules. The band gap of Ga-doped ZnO NPs is gradually narrowed with the increasing doping concentration, and a minimum value (3.16 eV) is reached with the Ga and Zn ratio of 3.8%, resulting in the maximum degree of CT. This work investigates the effects of doping induced band gap shrinkage on CT using SERS and provides a new insight on improving the SERS performance of semiconductor NPs.

## Introduction

The interfacial charge-transfer (CT) process between a substrate and an adsorbed molecule is an interesting phenomenon which has attached extensive research. The CT process has a wide range of applications in interface chemistry (Osako et al., 2018), catalytic chemistry (Thang et al., 2018), electronic devices (Liu et al., 2018), solar cells (Yadav et al., 2018), photoelectrochemistry (Chen et al., 2018), and so on. Therefore, it is vital to conduct an in-depth investigation of the CT process, which may provide us better understanding and thus expand its applications in various fields.

Surface-enhanced Raman scattering (SERS) is a forceful method for studying the CT between adsorbed molecule monolayer and the substrate (Wang et al., 2011; Li et al., 2018; Yu et al., 2018). As is well-known, the emergence of SERS phenomenon is inseparable from enhancement substrates. With the development of relevant researches, SERS active nanomaterials have been extended from noble metals, such as Au, Ag, and so on, to semiconductor nanomaterials (NMs) (Fleischmann et al., 1974; Tian et al., 2002; Biju et al., 2008; Shen et al., 2008; Alessandri and Lombardi, 2016). Many semiconductor NMs such as TiO_2_ (Yamada and Yamamoto, 1983), ZnO (Wang et al., 2009), NiO (Yamada et al., 1982), InAs/GaAs (Quagliano, 2004), CdS (Wang et al., 2007b), ZnS (Wang et al., 2007a) *etc*., have been demonstrated to exhibit good SERS activities. In comparison with metal substrates, semiconductor NMs have additional optical and electrical properties, which enable them to display remarkable CT enhancement and catalytic abilities (Lombardi and Birke, 2009; Han et al., 2017). As we all know, for the vast majority of semiconductors, their localized surface plasmon resonance is located in the infrared region. That is to say, when semiconductor NMs was used as enhancement substrate, SERS is mainly generated through the CT enhancement mechanism (Lombardi and Birke, 2007), so the SERS performance of semiconductor NMs is a powerful tool to investigate the CT process between the semiconductor NMs and adsorbed molecules.

The CT process between the adsorbed molecules and the substrate is closely related to the size and morphology of semiconductor NMs (Musumeci et al., 2009; Tang et al., 2012; Lamberti et al., 2015). Moreover, doping is also a very important factor for the CT process when semiconductor NMs are used as the SERS substrate. Our group have reported that the SERS performance of TiO_2_ nanoparticles (NPs) can be improved by Fe^3+^, Co^2+^, Ni^2+^ doping (Yang et al., 2011, 2014; Xue et al., 2013a), and the improvements were attributed to the formation of abundant doping (defects) levels in the band gap of TiO_2_ NPs. Dual functional Ta-doped electrospun TiO_2_ nanofibers with enhanced photocatalysis and SERS activity for detection of organic compounds were synthesized by Singh et al. (2017) The generation of Ti^3+^ defects from Ta^5+^ doping, which acted as an intermediate state for TiO_2_ to methylene blue molecules electron transfer, was used to explain the enhanced SERS activity of their obtained products. Effects of Mn^2+^, Zn^2+^, and Mg^2+^ doping on SERS properties of TiO_2_ NPs were also studied by our group (Yang et al., 2010; Xue et al., 2012a, 2013b), it was found that an appropriate amount of metal ions dopant enriched the surface states and improved the photo-generated carrier separation efficiency. SERS characteristics of Co^2+^ doped ZnO (Xue et al., 2012b) and Mn^2+^ doped CuO (Prakash et al., 2016) were also investigated, the influences of dopant were attributed to the increasing of defects and the ferromagnetic ordering, respectively. To date, most studies about the effect of metal doping semiconductor on SERS (CT process) focused on the change of surface state level or content of defects, however, the impact of doping induced band gap shrinkage on the CT process between semiconductor NPs and molecules have never been reported. For the first time, this paper investigates the effect of doping induced band gap shrinkage on the CT between semiconductor NPs and molecules.

Due to some unmatched properties in photoelectric and magnetism, ZnO is widely used in medicine and health care, food industry, varistor, antivirus, gas sensitive elements, and photocatalysis (Zhang et al., 2009; Cushen et al., 2012; Nohynek and Dufour, 2012; Hassan et al., 2013; Lang et al., 2014; Wang et al., 2014) etc. ZnO is also an important enhancement substrate in SERS study because of its wide band gap. Numerous SERS studies around ZnO NPs have been carried out. The size-dependent effect of ZnO NPs for SERS signal (Sun et al., 2007) and ZnO/PATP(p-aminothiophenol)/Ag assemblies (Sun et al., 2008) was investigated, the SERS enhancement mechanism for ZnO was attributed to the CT enhancement mechanism. The influence of contact variation in ZnO-molecules-metal system (Mao et al., 2012), fabrication of one-dimensional ZnO/4-MPy/Ag assemblies (Hu et al., 2010), contribution of ZnO to CT induced SERS in Au/ZnO/PATP assembly (Yang et al., 2008) and so on have been reported. Above all, the investigation about CT process between ZnO NPs and adsorbed molecules is necessary in view of the wide applications of ZnO NPs. Here we study the effect of doping induced band gap shrinkage on CT process between ZnO NPs and molecules. The band gap of ZnO NPs is altered by doping gallium (Ga) into ZnO NPs, the ionic radius of Ga^3+^ ions (0.062 nm) is less than the Zn^2+^ ions (0.074 nm), so the Ga^3+^ ions are soluble in the ZnO matrix.

In this work, a range of Ga-doped ZnO NPs with various ratio Ga and Zn and 4-MBA@ZnO (Ga-doped ZnO) system are obtained. The actual ratios of Ga and Zn in the Ga-doped ZnO NPs are confirmed by ICP measurement. Then it is determined that the degree of crystallinity and particles size of Ga-doped ZnO NPs can be affected by the ratio of Ga and Zn according to XRD, Raman, and TEM characterizations. Moreover, it is found that the band gap of NPs shrinks as the ratio of Ga and Zn increases. The effect of Ga doping on the CT process between ZnO NPs and 4-MBA monolayer is investigated using SERS. The modest amount of doped Ga can enhance the degree of CT between ZnO and 4-MBA monolayer compared to the pure ZnO NPs. The change of CT is mainly due to the size dependence effect and the band-gap shrinkage effect. The doped Ga causes the band gap shrinkage of ZnO and then affects the CT resonance process from the valence band (VB) of ZnO NPs to the LUMO of 4-MBA molecules. This work conducts an in-depth investigation on the effects of doping induced band gap shrinkage on CT using SERS and provides a new insight on improving the SERS performance of semiconductor NPs.

## Experimental Section

### Chemicals

4-Mercaptobenzoic acid (4-MBA) and Gallium(III) nitrate hydrate were purchased from Sigma-Aldrich and used without further purification. All other chemicals were acquired from Beijing Chemical Reagent Factory and used without further purification. The distilled and deionized water from a Milli-Q-plus system with the resistivity >18.0 MΩ was used in aqueous solution.

### Synthesis of ZnO and Ga-Doped ZnO NPs

ZnO and Ga-doped ZnO NPs were synthesized as follows. In short, 40 mL of 0.5 mol/L NaOH solution was slowly added dropwise into 100 mL of 0.1 mol/L Zn(Ac)_2_ solutions under vigorous stirring in order to produce the Zn(OH)_2_ precipitate. Subsequently, 1.2 g of NH_4_HCO_3_ powder was added. After stirring for 30 min, a semitransparent zinc carbonate hydroxide colloid was obtained. Then the colloid was centrifuged and rinsed three times with purified water and absolute ethyl alcohol in alternation and dried at 80°C. Thus, the precursor of a small crystallite Zn_5_(CO_3_)_2_(OH)_6_ was formed. Then, the as-prepared precursor was calcined at 550°C for 2 h to obtain the ZnO NPs.

The synthetic methods of Ga-doped ZnO NPs is similar with what we described above. The difference is that there is an opportune amount of Ga(NO_3_)_3_ (0.0003, 0.0005, 0.0007, 0.0009, 0.002, 0.004, 0.006, 0.008, and 0.01 mol/L, respectively) was added to the Zn(Ac)_2_ solutions to obtain Ga-doped ZnO NPs with difference ratio of Ga and Zn.

### Adsorption of Probing Molecules

ZnO and Ga-doped ZnO NPs surface-modified by molecules were obtained as follows: 20 mg of ZnO and Ga-doped ZnO nanocrystals were dispersed in 20 mL of 4-MBA (1 × 10^−3^ M) ethanol solution and the mixture was stirred for 6 h. Then, the precipitate was centrifuged and rinsed with absolute ethyl alcohol twice. ZnO and Ga-doped ZnO nanocrystals modified by 4-MBA were obtained.

### Sample Characterization

The crystal structure of ZnO sample was determined by X-ray diffraction (XRD) using a Siemens D5005 X-ray powder diffractometer with a Cu Kα radiation source at 40 kV and 30 mA. X-ray photoelectron spectra (XPS) were obtained by using a Thermo ESCALAB 250 spectrometer with an Mg Ka excitation (1253.6 eV). Elemental analysis was carried out by ICP-AES with an Agilent 725 spectrometer. The UV-Vis DRS spectra were recorded on a Shimadzu UV-3600 spectrophotometer. Transmission electron microscopy (TEM) images were taken using a JEM-2100F high-resolution transmission electron microscopy operating at 200.0 kV. Raman spectra acquired at ambient pressure were obtained by using a Horiba-Jobin Yvon LabRAM ARAMIS system with the resolution of ca. 4 cm^−1^; The 633 nm radiation from a 20 mW air-cooled HeNe narrow bandwidth laser was used as exciting source. The laser beam was focused onto a spot with a diameter of approximately 1 μm using an objective microscope with a magnification of 50×. The Raman band of the silicon wafer at 520.7 cm^−1^ was used to calibrate the spectrometer. Data acquisition was the result of two times 30 s accumulations for the 4-MBA molecules absorbed on ZnO (Ga-doped ZnO) NPs.

## Results and Discussion

### Measurement of XRD

**Figure 1:**
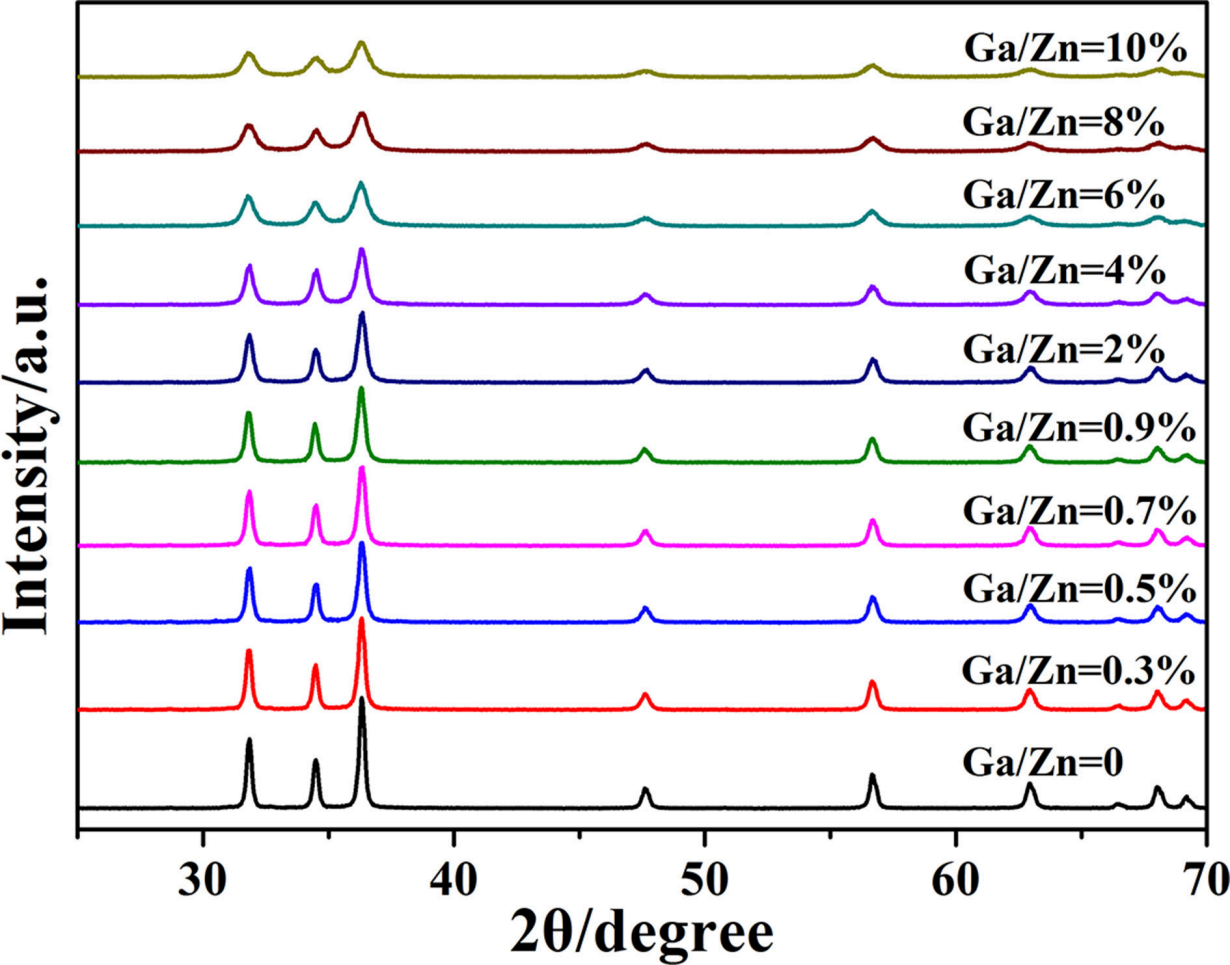
XRD spectra of ZnO and Ga-doped ZnO NPs with different ratio of Ga and Zn.

The XRD patterns of Ga-doped ZnO NPs with different ratio of Ga and Zn are shown in Figure 1. All the diffraction peaks of pure and doped ZnO are the characteristic peak of hexagonal wurtzite ZnO (JCPDS36-1451). No characteristic peaks corresponding to Ga or Ga_2_O_3_ are observed in the diffraction patterns, which is because the concentration of gallium is too low for those impurities to be detected by the XRD instrument. Figure S1 demonstrates the relationship between full width at half maximum (FWHM) of the peak (101) and the ratio of Ga and Zn : the intensity of the peak decreases and the FWHM is increased with the ratio of Ga and Zn increasing. All these results indicate that the Ga is doped into ZnO successfully and the crystallinity of Ga-doped ZnO NPs is decreased with the increasing doping ratio. The diameter of Ga-doped ZnO NPs (0–10%) are 35.4, 30.1, 27.4, 26.2, 25.0, 22.6, 19.0, 14.4, 13.5, and 12.9 nm, respectively, calculated using Scherrer's formula (Swamy et al., 2006) based XRD data.

### Measurement of XPS and ICP

According to the XPS spectra which is shown in Figure S2A, we can observe that all samples show similar characteristics as the pure ZnO spectra. However, in terms of Figure S2B, the relevant peaks of Ga-dopant (Ga 2p_1/2_ and Ga 2p_3/2_ located at 1117.9 and 1144.8 eV, respectively) can be observed when the spectra is magnified in the approximate range of 1110–1150 eV, moreover, the intensities of Ga-dopant-related peaks increases with the Ga concentration increasement. Figure S2C shows the XPS spectra of Zn_3/2_ and Zn_1/2_ for pure and doped ZnO NPs, and the two peaks are observed at 1021.7 and 1044.7 eV, respectively, the data is consistent with the binding energy of Zn-O (Sano et al., 2002; Jin et al., 2009).

All the description about the ratio of Ga and Zn used previously is the initial ratio, the ICP test is conducted in order to determine the actual Ga and Zn ratio of doped ZnO NPs. The results (initial ratio 0–10%) are 0, 0.29, 0.39, 0.56, 0.66, 1.4, 2.7, 3.8, 5.0, and 6.1%, respectively. It is clear that the results of quantitative analysis from ICP is similar with the initial ratio in the low doping ratio and a significant discrepancy appears when the initial ratio is higher than 0.9%. The reason for this phenomenon is that the vast majority of Ga is doped into the lattice of ZnO at low doping ratio, and a portion of Ga is not introduced into ZnO when the content of Ga is too high. Above all, the XPS measurement proves that the Ga is doped into ZnO successfully and the actual ratio of Ga and Zn is determined by ICP.

### Measurement of TEM Images and UV-vis Spectra

**Figure 2:**
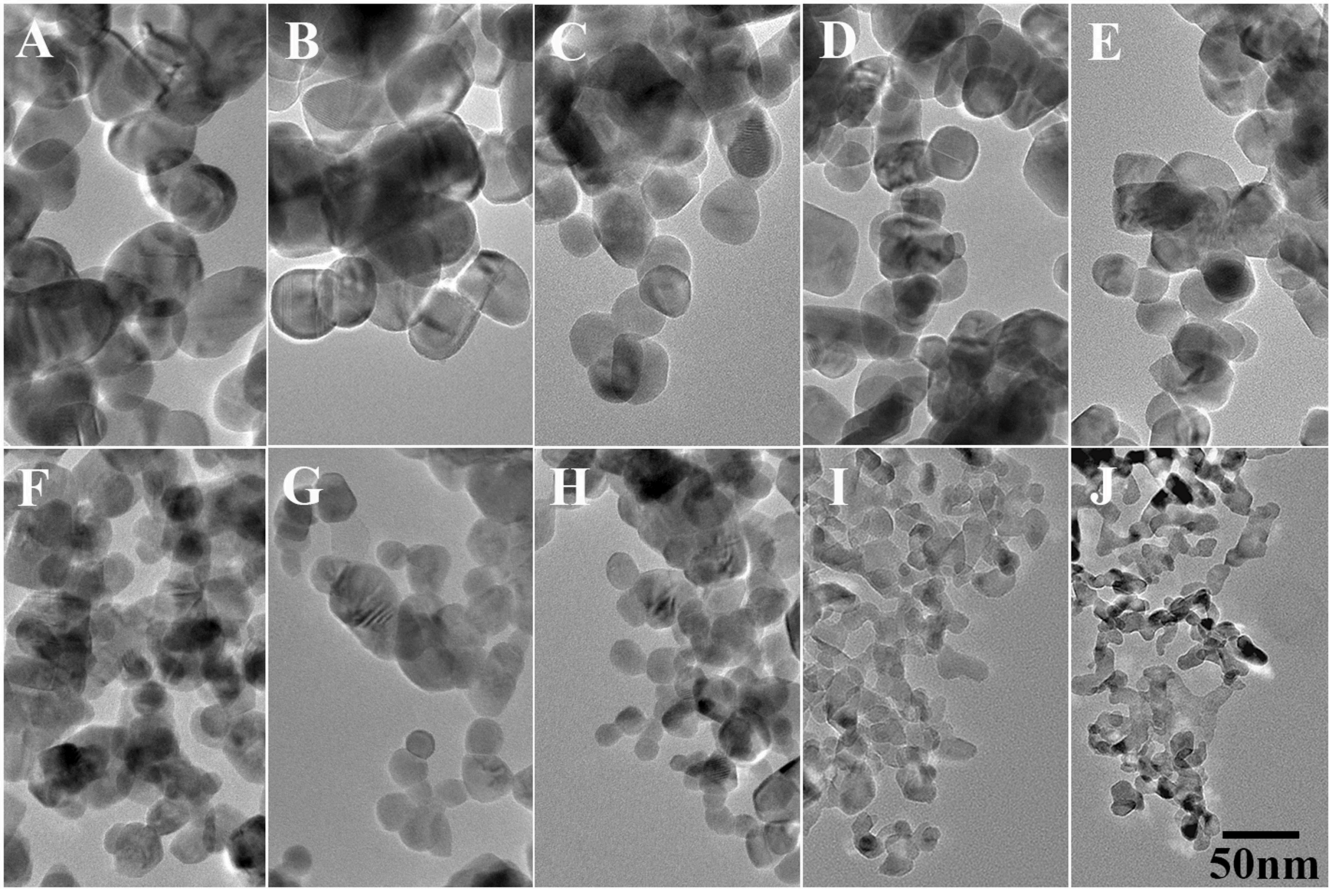
TEM images of Ga-doped ZnO NPs: **(A–J)** represent the initial ratio of 0, 0.3, 0.5, 0.7, 0.9, 2, 4, 6, 8, and 10% for the Ga-doped ZnO NPs, respectively. The bar is 50 nm, and it is common to all images.

Figure 2 shows the TEM images of pure and doped ZnO NPs, all the NPs are spherical and the diameters decreases with the increased Ga/Zn ratio. The particle sizes of the NPs are determined as approximate 51.4, 44.3, 35.7, 31.4, 28.6, 25.7, 22.9, 15.7, 14.3, and 12.9 nm, which are a little different from the value calculated from XRD. Such difference occurs due to the fact that the calculation results of XRD are based on the assumption that the materials are all signal crystals, and the measured values from TEM represent the size of nanoparticles which consist of one or multiple single crystal. However, the tendency of size change coincided with the result of XRD, evidencing that the particle diameter decreases as the doping concentration increases.

**Figure 3:**
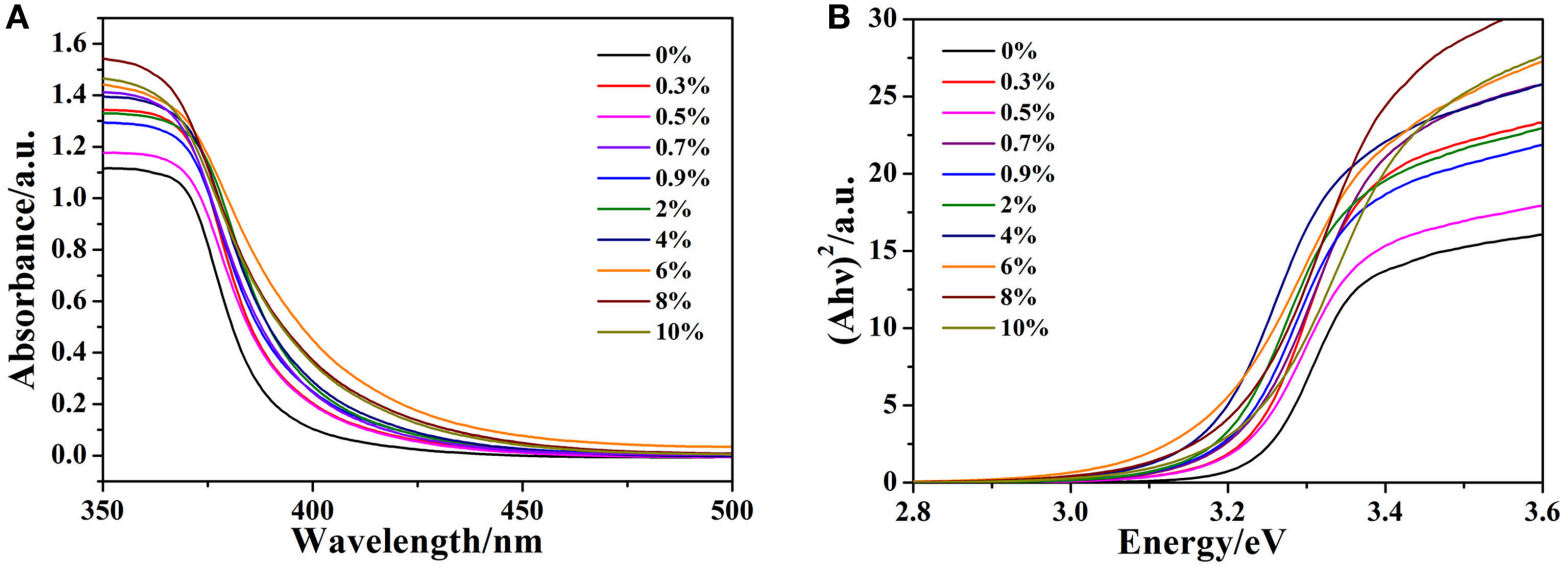
**(A)** The optical absorption spectra of pure and doped ZnO NPs, **(B)** Relationship between (A*h*ν)^2^ and photon energy (*h*ν) for Ga-doped ZnO NPs.

The effect of doped gallium on the optical properties of ZnO is investigated via UV-vis absorption spectroscopy. The optical absorption spectra are shown in Figure 3A. The steep drop of the absorption at about 378 nm is assigned to the CT process between valence band (VB) and conduction band (CB). Besides, all the absorption edge of Ga-doped ZnO NPs have a red shift compared to the pure ZnO and the maximal absorption edge appeared at the initial ratio of 6%. The phenomenon is attributed to the effect of doping on the carrier density. The band gap of ZnO NPs was calculated according to the absorption edge. Due to the fact that ZnO has a direct inter-band transition (Mahdhi et al., 2015) and on the basis of practical fact and theoretical calculation, the band gap of ZnO can be obtained through the Tuac's relation (Tauc, 1968; You and Hua, 2012):

(1) $$\begin{array}{l} A\left(h\nu \right)=\text{B}{\left(h\nu -{\text{E}}_{\text{g}}\right)}^{1/2} \end{array}$$

where A is the absorbance, *h*ν is the energy of the incident photon and E_g_ is the band gap. The band gap is determined by plotting (A*h*ν)^2^ vs. *h*ν and extrapolating the straight-line portion to the energy axis, the plots are shown in Figure 3B and the numerical values of E_g_ (0–10%) are 3.24, 3.22, 3.21, 3.20, 3.19, 3.18, 3.17, 3.16, 3.20, and 3.21 eV, respectively. According to this figure, with the increasement in doping concentration, the band gap of Ga-doped ZnO is first shrinked and down to the minimum value at the initial ratio of 6%, then increased. It should be noted that all the band gap of Ga-doped ZnO is less than the pure ZnO. Generally, there are two well-known theories about the changes of band gap of semiconductors: Burstein-Moss (BM) effect (Burstein, 1954; Moss, 1954) and band gap renormalization (BGR) effect (Dou et al., 1997; Jeon et al., 2011). The former is always related to the widening of the band gap and the latter is in connection with the shrinking-effect. The BGR effect is dominant in our system, the electron-impurity interactions, exchange interactions, and electron-electron Coulomb within the CB resulted in the shrinkage of the host band gap.

### Raman Spectra of ZnO and Ga-Doped ZnO NPs

**Figure 4:**
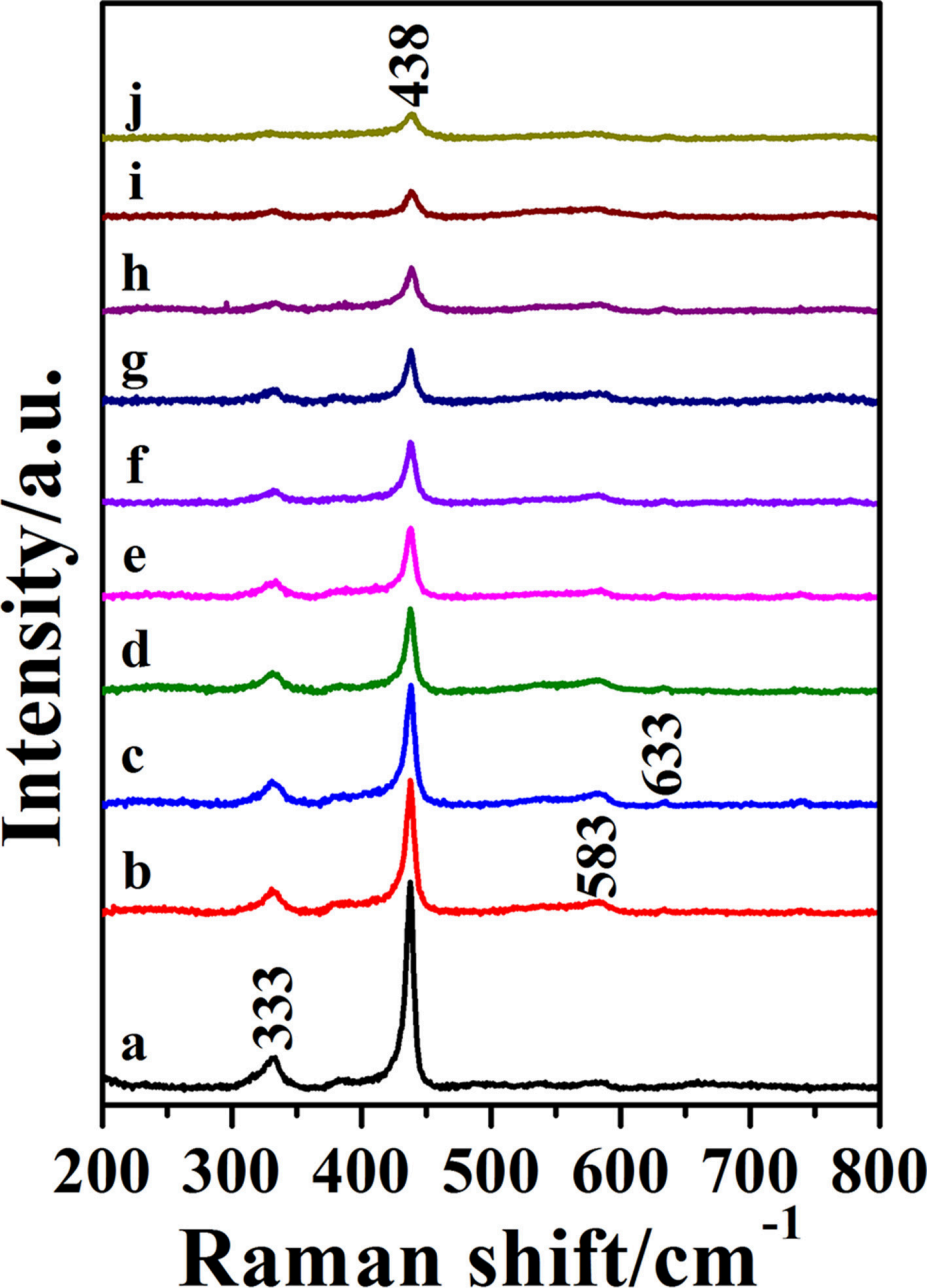
The Raman spectra of Ga-doped ZnO NPs with various initial ratio of Ga and Zn(from a to j): 0, 0.3, 0.5, 0.7, 0.9, 2, 4, 6, 8, and 10%.

The Raman spectra of pure and doped ZnO NPs are shown in Figure 4, all the Raman peaks are the characteristic peaks of hexagonal wurtzite structure. The ZnO NPs with hexagonal wurtzite phase possess C_6v_^4^ (P63/mc) space group and are simple single axial crystals. Only the optical phonon in the center of Brillouin zone is related to the first-order Raman scattering for perfect ZnO single crystal. In the point-group theory, the optical phonon modes are classified as Γ_opt_ = A_1_+2B_1_+E_1_+2E_2_, the A_1_ and E_1_ are split into transverse optical (TO) and longitudinal optical (LO) on account of they are polar modes. In all the phonon modes, A_1_ and E_1_ modes have Raman and infrared active, and that E_2_ modes only have Raman active, B_1_ modes are silent. According to the group theory and Figure 4, the peak at 333 cm^−1^ is the E_2_(high)-E_2_(low) mode and this mode is related to the multi-phonon scattering process. The strongest peak located at 438 cm^−1^ is the E_2_(high) mode. This is the characteristic peak of the wurtzite phase and its intensity is associated with the crystallinity of ZnO; the weak peak at 583 cm^−1^ is assigned to the A_1_(LO)-E_1_(LO) mode and the E_1_ vibrational mode is concerned with defects such as oxygen vacancy, zinc interstitial and the complex-defect and so on. The origin of another weak peak at 633 cm^−1^ is uncertain although it has been reported in ZnO films (Bundesmann et al., 2003; Shinde et al., 2011). Above all, all the peaks together prove the pure and doped ZnO NPs as hexagonal wurtzite phase, moreover, their crystallinity decrease with the ratio of Ga and Zn increasing, judging by the changes of E_2_(high) modes. All the results are consistent with the results of XRD and TEM.

### SERS Spectra of 4-MBA Adsorbed on Pure and Ga-Doped ZnO NPs

**Figure 5:**
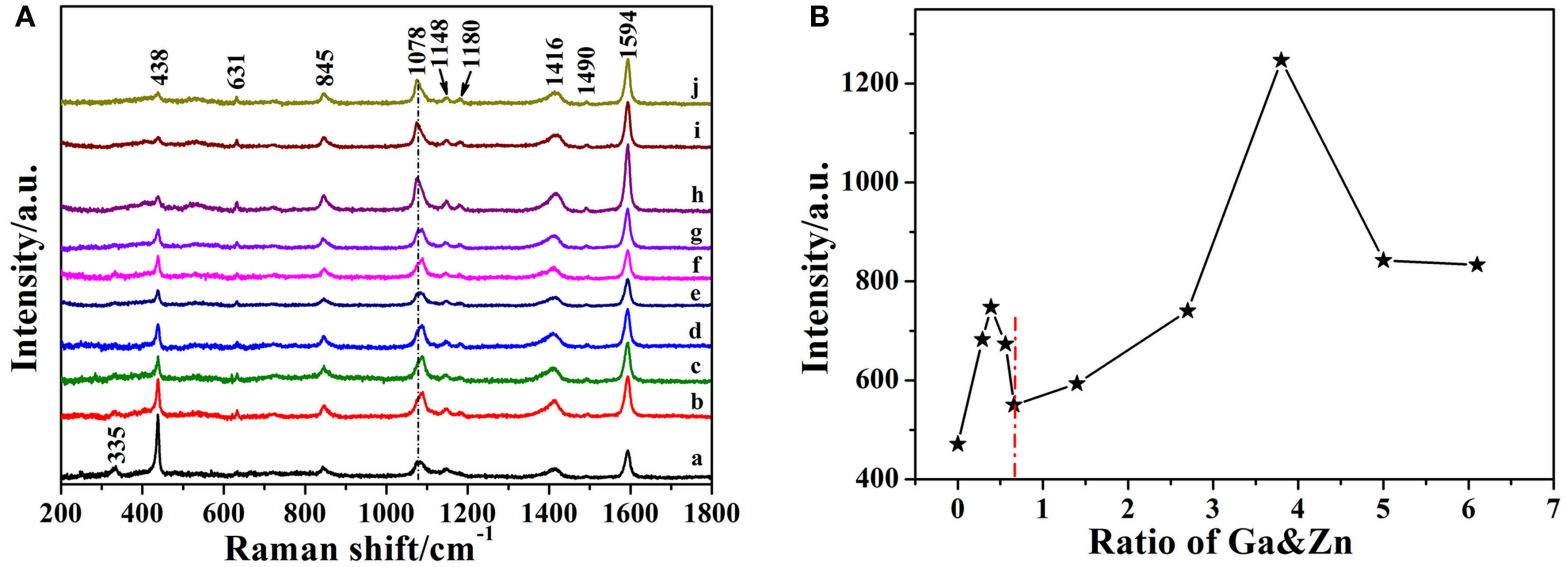
**(A)** SERS spectra of 4-MBA adsorbed on Ga-doped ZnO NPs with various initial ratio of Ga and Zn(from a to j): 0, 0.3, 0.5, 0.7, 0.9, 2, 4, 6, 8, and 10%. **(B)** The relationship between the intensity of the 1,594 cm^−1^ peak and the actual ratio of Ga and Zn for Ga-doped ZnO NPs.

The SERS spectra of 4-MBA adsorbed on pure and Ga-doped ZnO NPs are obtained as shown in Figure 5A, all the peaks are the characteristic peaks of 4-MBA adsorbed on ZnO NPs and coincides with the reported results from literature (Sun et al., 2007). The strong peaks located at 1,594 cm^−1^ and 1,078 cm^−1^ can be attributed to υ_8a_ (a1) and υ_12_ (a1) aromatic ring characteristic vibrations, respectively. It is worth mentioning that there is an irregular change in the peak position of 1,078 cm^−1^, the reason for this is attributed to the effect of the vibration mode of ZnO at 1,069 cm^−1^ (see the Figure S3). Other weak peaks such as 1,148 (υ_15_,b2) and 1,180 (υ_9_,a1) cm^−1^ are corresponded to the C-H deformation modes and agree well with the literature data (Sun et al., 2007; Xue et al., 2012b). The Raman shifts and assignments of the peaks are listed in Table 1. Moreover, it can be certified was that the 4-MBA molecules are bonded to the surface of ZnO (Ga-doped ZnO) by sulfhydryl; comparing the Raman spectrum of bulk 4-MBA with the SERS of 4-MBA adsorbed on ZnO NPs, the peaks at 913 cm^−1^ of 4-MBA powder disappeared when the molecules are adsorbed on the surface of ZnO. The relational graph between the intensity of the 1,594 cm^−1^ peak and the actual ratio of Ga and Zn is plotted and shown in Figure 5B, and an interesting phenomenon is observed. With the actual ratio increasing, the intensity increases to a first maximum value when the actual ratio is 0.39%, then the intensity decreases and down to the minimum value when the actual ratio is 0.66%. Interestingly, the intensity increases again and the second maximum value appears at the actual ratio of 3.8%. The reason for this phenomenon will be discussed in the next section. Moreover, to estimate and compare the enhancement ability of Ga-doped ZnO NPs with various ratio of Ga and Zn, the magnitude of the enhancement factor (EF) is calculated (see the Supporting Information). The EF of pure and Ga-doped (6%, initial ratio) ZnO NPs are 3.29 × 10^3^ and 8.13 × 10^3^, respectively, obviously demonstrating that doping Ga into ZnO NPs can significantly improve the enhancement ability of ZnO NPs. In other words, the degree of CT between ZnO NPs and 4-MBA monolayer is improved due to the Ga doping into ZnO NPs. The Ga doping induced band gap shrinkage can enhance the CT process between ZnO NPs and 4-MBA. The EF of Ga-doped ZnO NPs with other ratio is also calculated and is shown in the Table S1.

**Table 1:** Raman Shifts and Assignments of 4-MBA molecule adsorbed on ZnO (Ga-doped ZnO) NPs.

**Raman (bulk)**	**SERS (on ZnO)**	**Assignment**
1,596	1,594	υCC,8a(a1)
1,451	1,490	υCC+δCH
1,405	1,416	υCOO–
1,182	1,180	υCH,9(a1)
	1,148	υCH,15(b2)
1,098	1,078	υCC,12(a1)
913		δCSH,9b(b2)
813	845	δOCO

*δ = bend or deformation; ν = stretch*.

### Enhancement Mechanism and the Effect of Doped Ga on CT of ZnO NPs

**Figure 6:**
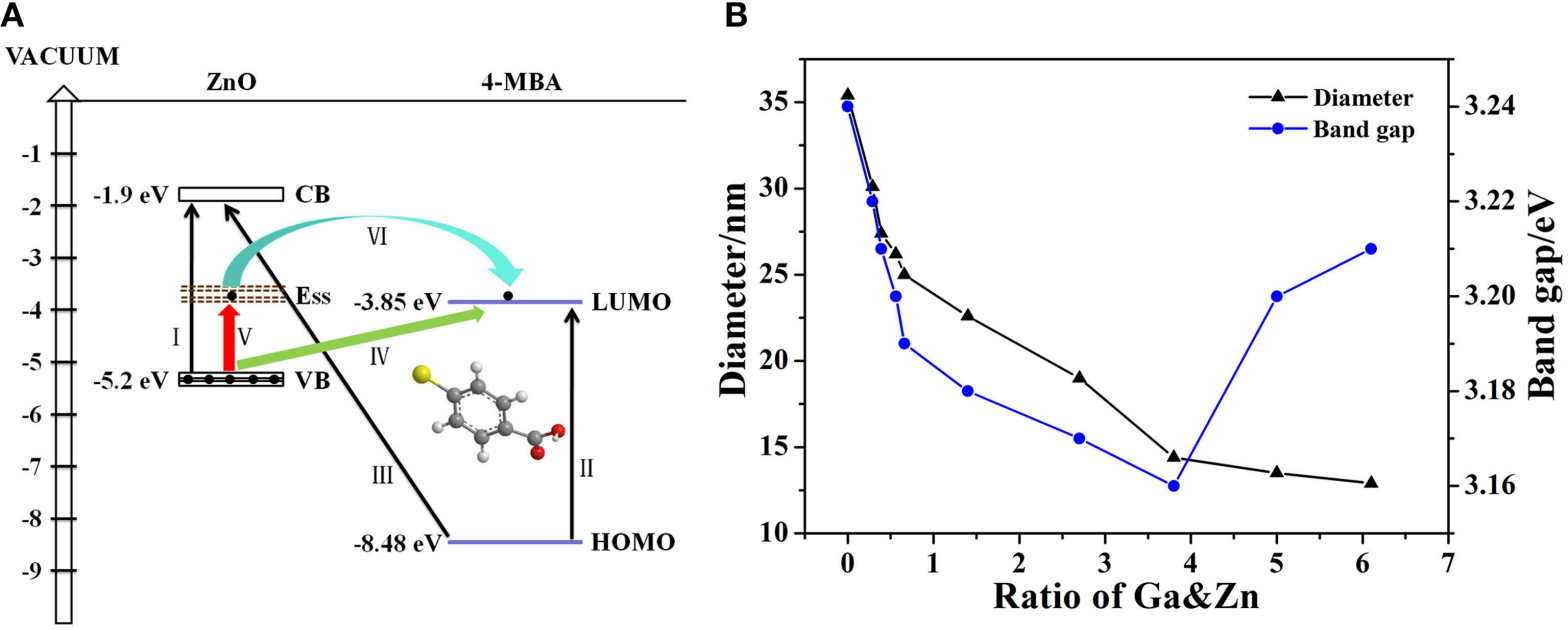
**(A)** All the possible CT resonance process in ZnO-molecules system, **(B)** The relationship between diameter, band gap and the actual ratio of Ga and Zn for Ga-doped ZnO NPs.

It is well-known that there are two main mechanisms widely accepted to interpret SERS: EM and CM. The former is related to the surface plasmon resonance (SPR) of the substrate, while the CT process is required for the latter. As we mentioned in the Introduction, the CT enhancement mechanism contributes to our system dominantly. In this system, the CT process occurs between ZnO NPs and the adsorbed 4-MBA molecules. All the possible CT resonance process in our ZnO-molecules system are shown in Figure 6A. The numerical values are referenced from literature data, such as: the highest occupied molecular orbit (HOMO) and the lowest unoccupied molecular orbit (LUMO) level of 4-MBA molecule are −8.48 and −3.85 eV (Yang et al., 2009), respectively; the CB and the VB level of ZnO NPs are −1.9 and −5.2 eV (Xue et al., 2012b), respectively. In addition, the Roman numerals in the figure represent different types of CT resonance: I is the exciton resonance of ZnO (Ga-doped ZnO) NPs; II is the molecule resonance of 4-MBA; III, IV, V, VI are the photon induced CT resonance from matches energy level between ZnO NPs and 4-MBA molecules. The excitation energy required when the process of exciton resonance (I) and molecule resonance (II) takes place is 3.3 and 4.63 eV, respectively. However, the excitation energy provided by 633 nm laser is only 1.96 eV. Therefore, the two processes of CT resonance are ruled out due to the provided energy is far less than the amount of energy needed. Likewise, the photon induced CT resonance between the HOMO level of 4-MBA molecules and the CB of ZnO NPs is impossible to happen owing to the process needs 6.58 eV. According to the literature data, the surface state level of ZnO NPs is located at −3.5 eV (Xue et al., 2012b) approximately, the energy needed for the excited transition of electrons in the VB of ZnO NPs to surface state level (V) and the LUMO orbit of 4-MBA molecules (IV) are 1.7 and 1.35 eV, respectively. The laser excitation energy is enough for the two photon-induced CT resonance process. With respect to the process that the electrons in the surface state level of ZnO injected into LOMO level of molecules (VI), it is occurred by oneself due to the LUMO level of 4-MBA molecules was slightly below the surface state level of ZnO NPs.

When it comes to the effect of doped Ga on CT of ZnO NPs, there are two changes on the properties of ZnO NPs: particle size and band gap. Correspondingly, there are two main factors that can affect the enhance ability of ZnO NPs for SERS: size dependence effect and band-gap shrinkage effect. Figure 6B shows the relationship between diameter, band gap and the actual ratio of Ga and Zn for Ga-doped ZnO NPs. Combined with Figures 5B, 6B, we think the size dependence effect is predominant when the actual ratio of Ga and Zn is below 0.66% and the strongest SERS signal is obtained when the diameter of Ga-doped ZnO NPs is 27.4 nm. The variation of the intensity of SERS signal on the particle size of ZnO derives from the resonance effect between the level of ionized receptor-exciton complex on the surface and the frequency of incident light. Moreover, the result we obtained is consistent with the conclusion that the maximum SERS signal appeared when the crystal size is 27.7 nm for pure ZnO NPs (Sun et al., 2007). When the actual doping ratio increases over 0.66%, the band-gap shrinkage effect becomes the predominant factor to affect the SERS performance (degree of CT) of ZnO NPs. The maximum shrinkage of bang gap is 0.08 eV in all the doping ratios, so the CT resonance process of I, II, III (described in Figure 6A) are still forbidden. However, the photon induced CT resonance (IV, V, VI) can be influenced by the decrease of band gap, thus promoting the CT process. The gap between VB and the surface state level of ZnO is decreased, and the gap between VB of ZnO and the LUMO level of molecules is also reduced. Therefore, the excited transition of electrons in the VB of ZnO to surface state level (V) or the LUMO level of molecules (IV) are much easier, hence the SERS performance (degree of CT) of ZnO is improved and a stronger SERS signal can be obtained. Besides, due to the decreases of gap between surface state level of ZnO NPs and the LUMO of 4-MBA molecules, the efficiency of electrons in the surface state level of ZnO injected into LUMO level of molecules (VI) is raised. Above all, the improvement of SERS signal (degree of CT) is because the photon induced CT resonance (IV, V, VI) is enhanced due to band-gap shrinkage effect. Moreover, the reliability of the schematic diagram of the possible CT resonance process in our ZnO-molecules system can also be testified by the SERS spectra obtained at excitations of 532 and 785 nm, the data is shown in Figure S5.

## Conclusions

In summary, we synthesized a series of Ga-doped ZnO NPs with various ratio of Ga and Zn by a simple method and obtained the 4-MBA@ZnO system by modifying ZnO used 4-MBA molecules. The XRD and TEM measurements are carried out, the particle size diminishes gradually with the increasing ratio of Ga and Zn. When Ga is introduced into ZnO, the degree of crystallinity is decreased according to the results from XRD and Raman. Moreover, a Ga doping induced band gap shrinkage occurs: the band gap of Ga-doped ZnO narrows with the ratio of Ga and Zn increasing, and the band gap is down to the minimum value (3.16 eV) when the actual ratio of Ga and Zn is 3.8%. The last but not the least, the SERS performance (degree of CT) of Ga-doped ZnO NPs is investigated, and the doped Ga is found to enhance the intensity of SERS signal because it can cause the band gap shrinkage and then affect the CT resonance process. The band gap shrinkage can promote the photon induced CT resonance process, the electrons in the VB of ZnO NPs were excited transition to surface state level of ZnO NPs and then injected into the LUMO level of molecules, or that the electrons in the VB of ZnO NPs were directly excited transition into the LUMO level of molecules. In conclusion, this work is not only beneficial for in-depth understanding of the effect of doping on CT resonance process between adsorbed molecules and semiconductor, but also provides a new insight on improving the SERS performance of semiconductor NMs.

## Data Availability

All datasets generated for this study are included in the manuscript and/or the supplementary files.

## Author Contributions

PL performed the experiments and analyzed the date with help from BZ, XY, XW, XZ, and LZ. PL wrote and revised the manuscript with input from all authors. All authors read and approved the manuscript.

### Conflict of Interest Statement

The authors declare that the research was conducted in the absence of any commercial or financial relationships that could be construed as a potential conflict of interest.
