# Supplementary material for: Investigation of the Charge-Transfer Between Ga-Doped ZnO Nanoparticles and Molecules Using Surface-Enhanced Raman Scattering: Doping Induced Band-Gap Shrinkage
Source: Front Chem. 2019 Mar 19;7:144. doi: 10.3389/fchem.2019.00144 (PMC6433776; doi:10.3389/fchem.2019.00144)
Supplement: Supplementary file 1 [file Data_Sheet_1.docx]

Supplementary Material

Investigation of the Charge-transfer between Ga-doped ZnO Nanoparticles and Molecules Using Surface-enhanced Raman Scattering: Doping Induced Band-gap Shrinkage

Peng Li^1,2^, Xiaolei Wang^1,2^, Xiaolei Zhang^1,2^, Lixia Zhang^2^, Xuwei Yang^2*^ and Bing Zhao^1,2*^

^1^State Key Laboratory of Supramolecular Structure and Materials, Jilin University, Changchun, People’s Republic of China

^2^College of Chemistry, Jilin University, Changchun, People’s Republic of China

*** Correspondence:**Prof. Bing Zhao
[zhaob@jlu.edu.cn](mailto:zhaob@jlu.edu.cn)


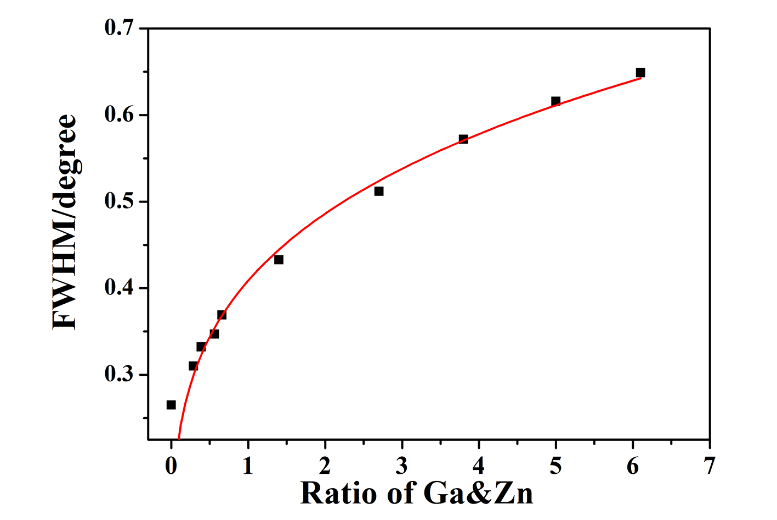


**Figure S1.** The relationship between FWHM of XRD and the ratio of Ga&Zn.


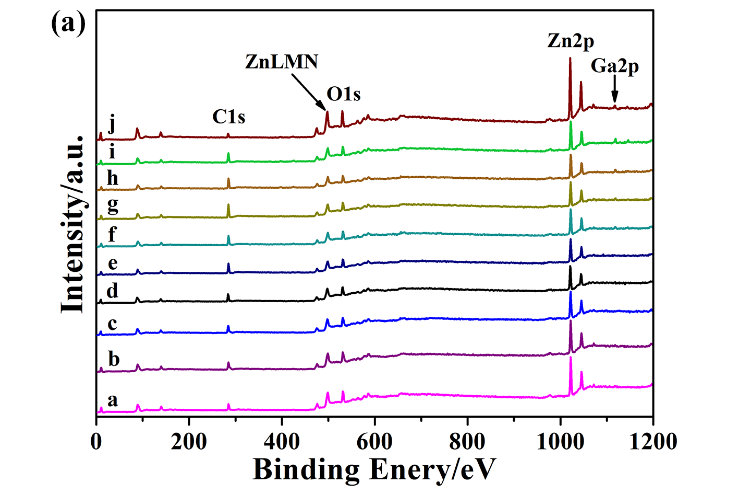

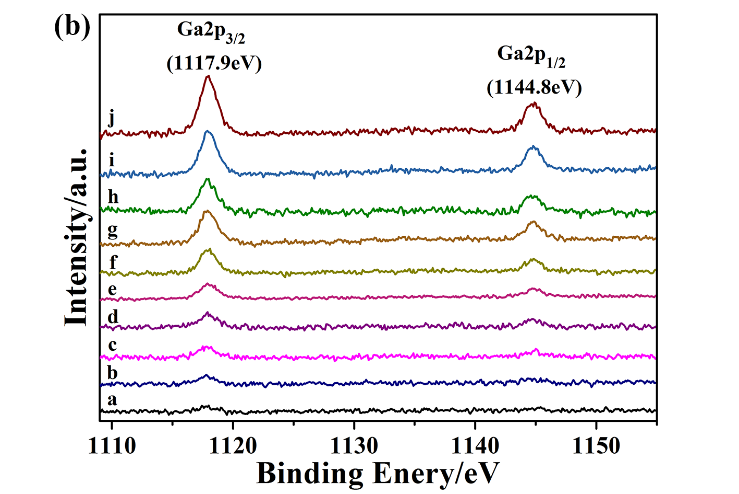

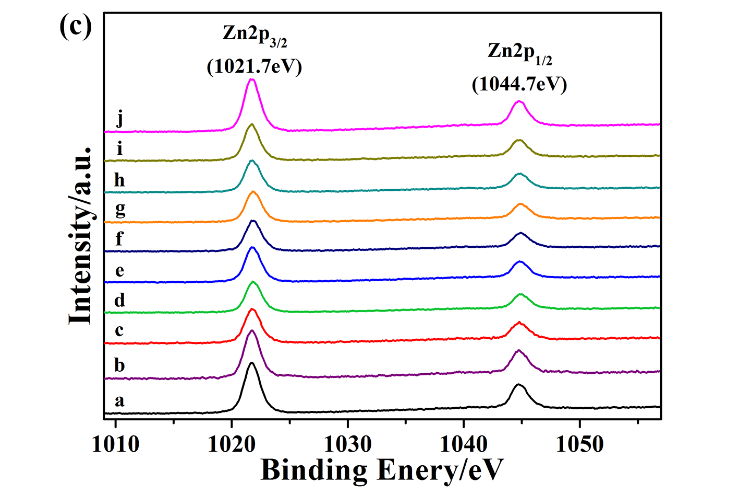


**Figure S2.** XPS spectra of ZnO and Ga-doped ZnO NPs with different ratio of Ga&Zn: (a) Wide scan, (b) Narrow scan at Ga2p_3/2_ and Ga2p_1/2_, (c) Narrow scan at Zn2p_3/2_ and Zn2p_1/2_ peak.


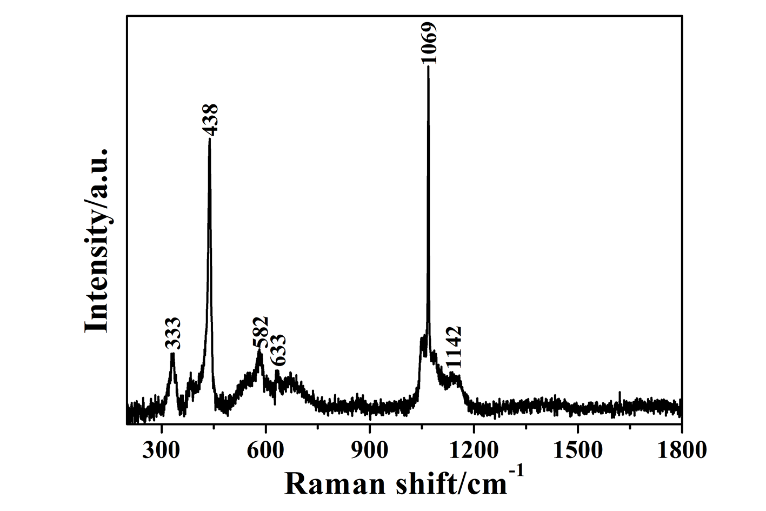


**Figure S3.** The Raman spectra of pure ZnO NPs in the range of 200-1800cm^-1^.


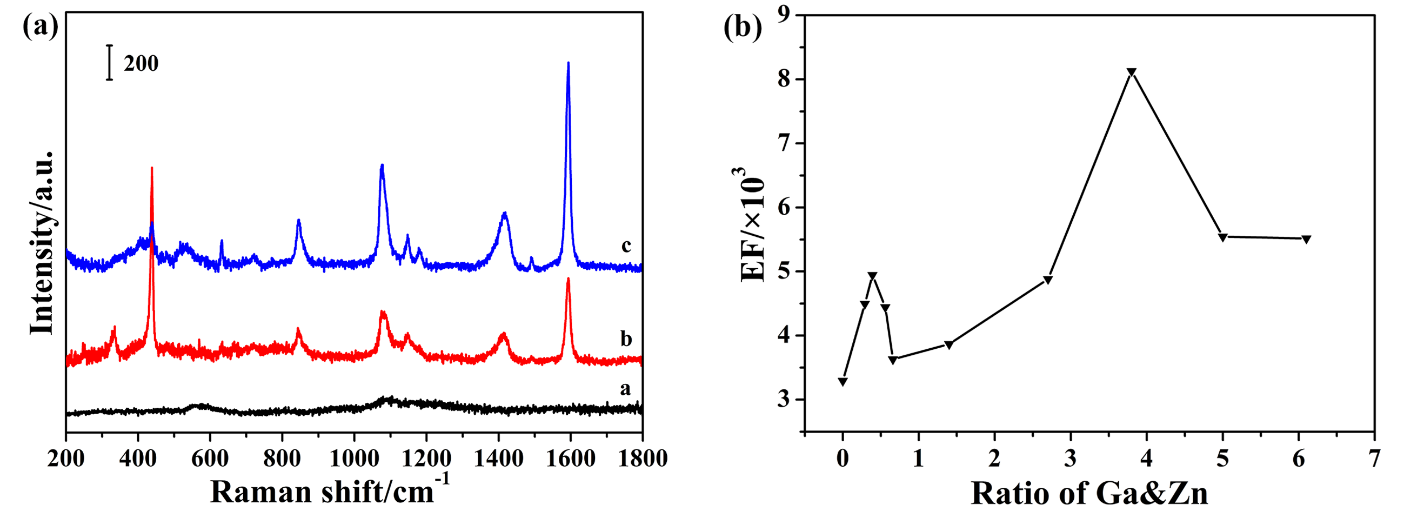


**Figure S4.** (a) a, b, c are the Normal Raman spectra of 4-MBA solution (0.1mol/L) and SERS spectra of 4-MBA adsorbed on pure and Ga-doped (6%, initial ratio) ZnO nanoparticles, respectively. (b) The relationship between the EF (calculated by intensity of the 1594 cm^-1^ peak) and the actual ratio of Ga&Zn for Ga-doped ZnO NPs.

**The enhancement factor (EF) of the Ga-doped ZnO nanoparticles.**

To estimate and compare the enhancement ability of Ga-doped ZnO NPs with various ratio of Ga&Zn accurately, we calculated the magnitude of the enhancement factor (EF) by the following equation (Xie et al., 2011; Zhao et al., 2015):

EF=(I_SERS_/N_SERS_)/(I_bulk_/N_bulk_)

Where I_SERS_ and I_bulk_ are the SERS intensities of 4-MBA on the Ga-doped ZnO NPs and normal Raman scattering intensity of the 4-MBA solution (0.1 mol/L) which was dropped (10μL) onto the glass and dried become a round spot, respectively. N_SERS_ and N_bulk_ are the numbers of the 4-MBA molecule adsorbed on the Ga-doped ZnO NPs and bulk molecule illuminated by the 633 nm laser excitation to obtain the corresponding SERS and normal Raman spectra, respectively. In our experiment, the laser spot is 1 μm in diameter and the penetration depth is 17 μm of the focused laser beam used; the diameter of round spot of 4-MBA solution is 3 cm; therefore, the N_bulk_ were calculated to be 6.69×10^8^. The mass of Ga-doped ZnO NPs in our experiment was 20 mg and the concentration and volume of the 4-MBA solution which was used to modify the Ga-doped ZnO NPs were 10^-3^ mol/L and 20 mL, respectively; the density of Ga-doped ZnO NPs is 5.606 g/cm^-3^; So, the number of molecules for SERS signal (N_SERS_) was calculated as 1.01×10^7^. The I_SERS_ and I_bulk_ were determined by the intensity of 1594 cm^-1^ peak in the Figure S4a, the EF of pure and Ga-doped (6%, initial ratio) ZnO NPs were 3.29×10^3^ and 8.13×10^3^, moreover, what we should note is that the EF was underestimated due to the number of molecules for SERS signal (N_SERS_) was overrated. The EF of Ga-doped ZnO NPs with other ratio was also calculated by the same method and shown in the Table S1. The relationship between the EF and the actual ratio of Ga&Zn of Ga-doped ZnO nanoparticles was shown in Figure S4b, the change tendency of EF was completely consistent with the change tendency of the intensity of the 1594 cm^-1^ peak which was shown in Figure 5b.

**Table S1.** The EF of Ga-doped ZnO NPs with various ratio of Ga&Zn.

| Initial ratio (%) | 0 | 0.3 | 0.5 | 0.7 | 0.9 | 2 | 4 | 6 | 8 | 10 |
| --- | --- | --- | --- | --- | --- | --- | --- | --- | --- | --- |
| Actual ratio (%) | 0 | 0.29 | 0.39 | 0.56 | 0.66 | 1.4 | 2.7 | 3.8 | 5.0 | 6.1 |
| EF (×10^3^) | 3.29 | 4.49 | 4.96 | 4.44 | 3.43 | 3.87 | 4.88 | 8.13 | 5.55 | 5.52 |


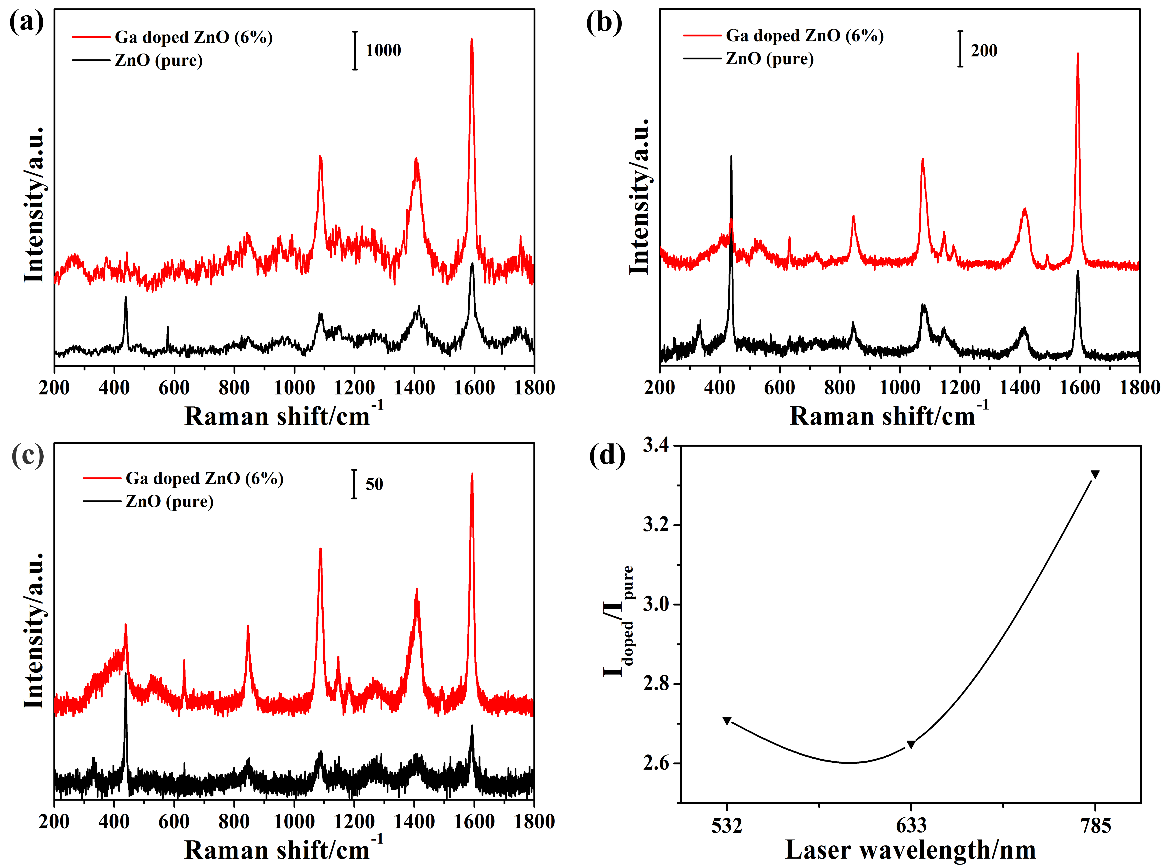


**Figure S5.** Raman spectra of 4-MBA adsorbed on Ga-doped ZnO (3.8%, actual ratio) and pure ZnO NPs, the date was obtained at excitations of: (a) 532, (b) 633, (c) 785 nm; (d) The relationship between I_doped_/I_pure_ and laser wavelength, I_doped_ and I_pure_ are the intensity of 1594 cm^-1^ when the molecules adsorbed on Ga-doped ZnO (3.8%, actual ratio) and pure ZnO NPs, respectively.

**The SERS spectra of Ga-doped ZnO nanoparticles under various laser wavelength.**

According to Figure S5(a,b,c), we can see that the absolute intensity of SERS signal were decreased with the laser wavelength increased whether the molecules adsorbed on the Ga-doped or pure ZnO NPs. Then we plotted the I_doped_/I_pure_ (I_doped_ and I_pure_ are the intensity of 1594 cm^-1^ when the molecules adsorbed on Ga-doped ZnO (3.8%, actual ratio) and pure ZnO NPs, respectively) versus laser wavelength as shown in Figure S5d, the specific value at 532 and 633 nm is similar, however, it was increased at the 785 nm. The interesting phenomenon is certainly proved the validity of the schematic diagram of the possible charge transfer (CT) resonance process in our ZnO-molecules system presented at Figure 6a. The excitation energy of 532, 633 and 785 nm are 2.33, 1.96, and 1.58 eV, respectively. In Figure 6a, the energy required for resonance process Ⅳ and Ⅴ are 1.35 and 1.70 eV (molecules adsorbed on pure ZnO NPs), so the excitation energy of 532 and 633 nm are enough for the two process while the excitation energy of 785 nm can only make sure the process Ⅳ happens and it is insufficient for the process Ⅴ. The band gap of Ga-doped ZnO is narrowing when we introduced the Ga into ZnO NPs, and meanwhile the difference value between the valence band and surface state level of Ga-doped ZnO is decreased. So, the I_doped_/I_pure_ was significantly increased at excitations of 785 nm due to the excitation energy of 785 nm is adequate for the resonance process Ⅳ and Ⅴ now. Hence, the reliability of the schematic diagram (Figure 6a) of the possible CT resonance process in our ZnO-molecules system was testified by the SERS spectra obtained at excitations of 532, 633 and 785 nm.

**References**

Xie, Y., Wang, X., Han, X., Song, W., Ruan, W., Liu, J., et al. (2011). Selective SERS detection of each polycyclic aromatic hydrocarbon (PAH) in a mixture of five kinds of PAHs. *J. Raman Spectrosc.* 42(5)**,** 945-950. doi: 10.1002/jrs.2818.

Zhao, H., Jin, J., Tian, W., Li, R., Yu, Z., Song, W., et al. (2015). Three-dimensional superhydrophobic surface-enhanced Raman spectroscopy substrate for sensitive detection of pollutants in real environments. *J. Mater. Chem. A* 3(8)**,** 4330-4337. doi: 10.1039/c4ta06590e.
